# Specific Molecular Signatures for Type II Crustins in Penaeid Shrimp Uncovered by the Identification of Crustin-Like Antimicrobial Peptides in *Litopenaeus vannamei*

**DOI:** 10.3390/md16010031

**Published:** 2018-01-16

**Authors:** Cairé Barreto, Jaqueline da Rosa Coelho, Jianbo Yuan, Jianhai Xiang, Luciane Maria Perazzolo, Rafael Diego Rosa

**Affiliations:** 1 Laboratory of Immunology Applied to Aquaculture, Department of Cell Biology, Embryology and Genetics, Federal University of Santa Catarina, Florianópolis 88040-900 SC, Brazil; cairebarreto@gmail.com (C.B.); jaquesombrio@gmail.com (J.d.R.C.); l.m.perazzolo@ufsc.br (L.M.P.); 2 Key Laboratory of Experimental Marine Biology, Institute of Oceanology, Chinese Academy of Sciences, Qingdao 266071, China; yuanjb@qdio.ac.cn (J.Y.); jhxiang@qdio.ac.cn (J.X.)

**Keywords:** invertebrate immunity, host defense peptide, crustacean, WAP domain, molecular diversity, host-pathogen interaction

## Abstract

Crustins form a large family of antimicrobial peptides (AMPs) in crustaceans composed of four sub-groups (Types I-IV). Type II crustins (Type IIa or “Crustins” and Type IIb or “Crustin-like”) possess a typical hydrophobic N-terminal region and are by far the most representative sub-group found in penaeid shrimp. To gain insight into the molecular diversity of Type II crustins in penaeids, we identified and characterized a Type IIb crustin in *Litopenaeus vannamei* (Crustin-like *Lv*) and compared Type II crustins at both molecular and transcriptional levels. Although *L. vannamei* Type II crustins (Crustin *Lv* and Crustin-like *Lv*) are encoded by separate genes, they showed a similar tissue distribution (hemocytes and gills) and transcriptional response to the shrimp pathogens *Vibrio harveyi* and White spot syndrome virus (WSSV). As Crustin *Lv*, Crustin-like *Lv* transcripts were found to be present early in development, suggesting a maternal contribution to shrimp progeny. Altogether, our in silico and transcriptional data allowed to conclude that (1) each sub-type displays a specific amino acid signature at the C-terminal end holding both the cysteine-rich region and the whey acidic protein (WAP) domain, and that (2) shrimp Type II crustins evolved from a common ancestral gene that conserved a similar pattern of transcriptional regulation.

## 1. Introduction

Crustins are cysteine-rich antimicrobial peptides (AMPs) holding a typical whey acidic protein (WAP) domain [1]. The WAP domain is a conserved motif containing eight cysteine residues, forming a characteristic four-disulfide core (4DSC) arrangement, that is also found in many proteins exhibiting protease inhibitory properties or regulatory functions in growth and tissue differentiation [2]. These bioactive molecules were originally isolated from the hemolymph of the shore crab *Carcinus maenas* as a cationic 11.5-kDa peptide with specific activity against marine Gram-positive bacteria [3]. Homologues of the *C. maenas* 11.5-kDa peptide were identified some years later in two shrimp species (*Litopenaeus vannamei* and *Litopenaeus setiferus*) and tentatively named as “Crustins” [4], a nomenclature that was subsequently accepted and adopted for this AMP family [1,5,6,7]. Indeed, crustins comprise a large and diverse family of gene-encoded AMPs in decapod crustaceans [8], but they are also present in non-decapod species, such as amphipods, copepods and isopods, and even in some hymenopteran insects [1].

Based on the presence or absence of structural domains lying at the N-terminal region, Smith et al. [5] classified crustins into three sub-groups, designated as Types I to III. Type I crustins are most related to the *C. maenas* 11.5-kDa peptide (later termed as “carcinin” [9]) and are characterized by the presence of four conserved cysteine residues, the cysteine-rich domain, upstream to the C-terminal WAP domain. They occur mainly in Pleocyemata (crabs, lobsters and crayfish), but some members were also reported in penaeid shrimp [10,11]. On the other hand, Type II crustins are mainly present in penaeids (Dendrobranchiata) and harbor a hydrophobic region rich in glycine residues, the glycine-rich domain, positioned at the N-terminal end of the cysteine-rich domain that is also found in Type I crustins. By contrast, Type III crustins (also known as single WAP domain-containing proteins or SWD) are only composed of a single WAP domain, and display both antimicrobial and antiprotease activities [12]. Proteins containing two WAP domains (double WAP domain-containing proteins or DWD) and crustin homologues from hymenopteran insects have been recently classified as Type IV and Type V, respectively [13].

Due to the economic importance of penaeid shrimp worldwide, Type II is the most well characterized sub-group of crustins. They are usually active against Gram-positive bacteria, but in vivo studies using gene silencing revealed that Type II crustins play a key role in shrimp defense against pathogenic Gram-negative bacteria [14,15,16]. Interestingly, the knockdown of Type II crustins in the Pacific white shrimp *L. vannamei* causes an increase in mortality after infections with the bacterial pathogen *Vibrio penaeicida*, but not in response to the fungal pathogen *Fusarium oxysporum* [14]. The antibacterial activity of Type II crustins appears to be related to the WAP domain. Indeed, it has been shown that Type II crustins with an incomplete WAP domain have impaired antimicrobial activities [17]. This large crustin sub-group showed to be diverse in terms of both structure and function. For instance, in the black tiger shrimp *Penaeus monodon*, at least ten different Type II crustins (crustin*Pm*1-10) have been identified, displaying diverse molecular structures and biological activities [7]. In the literature, this heterogeneous and complex crustin Type has been also subdivided into two sub-types, namely Type IIa or “Crustins”, most related to the first glycine-rich peptides identified in *Litopenaeus* species (Crustin *Lv* and Crustin *Ls*) [4], and Type IIb or “Crustin-like”, homologues of the Cru*Fc* peptide from *Fenneropenaeus chinensis* [17] and the Crus-like*Pm* (or crustin*Pm*7) peptide from *P. monodon* [18]. Interestingly, while Type IIa crustins have been identified in different penaeids [4,19,20,21], Type IIb crustins were only reported in Oriental (Asian) species [16,17,18,22].

Although a distinction between Type IIa and Type IIb crustins has been previously established (based on differences in the amino acid length between the cysteine-rich and WAP domains) [7], the current classification of these sub-types in penaeid shrimp remains confused and controversial, leading to misleading categorizations. In order to explore and refine this issue, we have firstly identified and characterized at both molecular and transcriptional levels a Type IIb crustin from the most commonly cultivated penaeid shrimp (*L. vannamei*), and then provided amino acid signatures specific to Type IIa and Type IIb crustins in penaeid shrimp. By taking advantage of publicly accessible databases, we have identified seven nucleotide sequences in *L. vannamei* transcriptomes showing high similarities to Type IIb crustins that were opportunely named Crustin-like *Lv*. *L. vannamei* Type IIa and Type IIb crustins are encoded by distinct genomic sequences and differ not only in the length of their structural domains, but with each sub-type possessing a specific amino acid signature at the C-terminal region containing the 12 conserved cysteine residues. A comparative gene expression analysis showed that *L. vannamei* Type IIa (Crustin *Lv*) and Type IIb (Crustin-like *Lv*) crustins are mainly expressed in circulating hemocytes and gills of juveniles and displayed a similar transcriptional pattern in response to two unrelated shrimp pathogens, the Gram-negative *Vibrio harveyi* and the White spot syndrome virus (WSSV). Finally, as observed for Crustin *Lv*, Crustin-like *Lv* transcripts were detected in all stages of *L. vannamei* development, from fertilized eggs to larval and postlarval stages. We provided here the first molecular characterization of a Type IIb crustin in an Occidental (non-Asian) species and the identification of molecular amino acid signatures specific to Type II crustins in penaeid shrimp.

## 2. Results and Discussion

### 2.1. Identification and Molecular Characterization of Type IIb Crustins in L. vannamei

**Figure 1:**
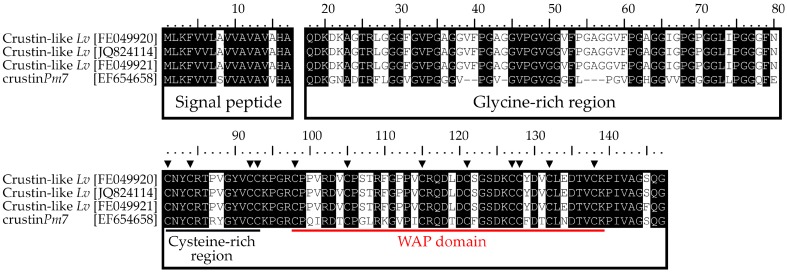
*Litopenaeus vannamei* Type IIb crustins. Amino acid sequences alignment of Type IIb crustins from *L. vannamei* (Crustin-like *Lv*) and the crustin*Pm*7 from *Penaeus monodon*. Identical residues are highlighted in black. Triangles (▼) indicate the 12 conserved cysteine residues found in crustins. The whey acidic protein (WAP) domain is underlined by a solid red line.

By taking advantage of publicly accessible databases, we have characterized for the first time Type IIb crustins in the most commonly cultivated penaeid shrimp, *L. vannamei*. In silico analysis led to the identification of three complete (one from the GenBank Nucleotide database and two from Expressed Sequence Tag libraries) and four incomplete/partial (Transcriptome Shotgun Assembly database) nucleotide sequences. All sequences corresponded to Type IIb crustins that were tentatively named Crustin-like *Lv*. *L. vannamei* Type IIa crustins were previously termed as Crustin *Lv* [4]. The complete full-length cDNA sequences (GenBank: JQ824114, FE049920 and FE049921) encode 147 amino acid precursors, starting with a predicted 17-residue signal peptide followed by a putative cationic (p*I* ~8) mature peptide of 130 amino acid residues (Figure 1). The presence of this signal peptide is necessary to direct the precursors to the endoplasmic reticulum and the Golgi apparatus. Then, the mature peptides are targeted to intracellular granules where they are stored [23]. All mature peptides possess a glycine-rich region containing three repeats of the pentapeptide Val-Phe-Pro-Gly-Ala (VFPGA) at the N-terminal end, followed by a C-terminal region containing twelve conserved cysteine residues, eight of them comprising a single WAP domain (Figure 1). For the incomplete Crustin-like *Lv* sequences (GenBank: GETD01016911, HAAW01014776, GETZ01011995 and GDUV01030786), only the C-terminal region, holding both the cysteine-rich region and the WAP domain, was identified. These incomplete sequences lack the signal peptide and part of the glycine-rich region (Figure S1).

Crustin-like *Lv* deduced amino acid sequences were compared with Type II crustins from penaeid shrimp and with crustins from other crustacean species (Figure S1). Mature Crustin-like *Lv* sequences shared a high amino acid sequence identity with other Type IIb crustins, such as Crustin-like from *Marsupenaeus japonicus* (75%; [16]), the *Fi*-crustin from *Fenneropenaeus indicus* (73%; [22]), crustin*Pm*7 (Crus-like*Pm*) from *P. monodon* (70%; [18]) and Cru*Fc* from *F. chinensis* (69%; [17]). Within Type IIa crustins, Crustin-like *Lv* was 44% identical to Crustin *Lv* sequences [4] and 40% to 54% identical to other Type IIa crustins from penaeids [10,19,20,21]. Regarding Type II crustins from non-penaeid species (Pleocyemata), Crustin-like *Lv* showed 43% to 51% amino acid identity with the crustins (PJC1 to PJC4) from the Japanese spiny lobster *Panulirus japonicus* [24]. Finally, *L. vannamei* Type IIb sequences showed less identity with the other crustins sub-groups found in crustaceans, namely Type I (30–43%), Type III (20–25%), and Type IV (25–27%).

### 2.2. L. vannamei Type II Crustins Are Encoded by Distinct Genes

**Figure 2:**
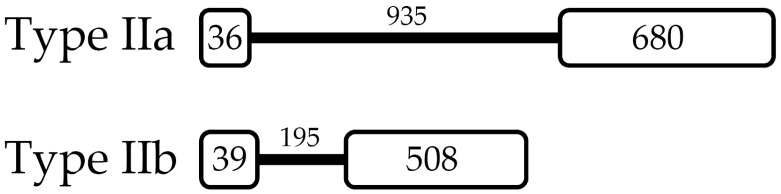
*Litopenaeus vannamei* Type II crustins are encoded by distinct genomic sequences. A not-to-scale representation of *L. vannamei* Type II crustins, Type IIa (Crustin *Lv*) and Type IIb (Crustin-like *Lv*). White boxes indicate the exons and the black line indicates the intron. The numbers show the size (in base pairs) of the exons and introns.

The *L. vannamei* Type IIa gene that encodes Crustin *Lv* is 1651 bp in size and consists of two exons (36 bp and 680 bp, respectively) interrupted by a single intron (935 bp) (Figure S2). The first exon covers the 5′ untranslated region (5′-UTR) and only two codons of the signal peptide (ATGAAG), while the second exon encodes the remainder of the signal peptide sequence, and the complete mature peptide and the 3′ untranslated region (3′-UTR) (Figure 2 and Figure S2). Likewise, the Type IIb gene (742 bp) (Figure S2), that encodes Crustin-like *Lv*, is also composed of two exons (39 bp and 508 bp, respectively) separated by one intron (195 bp). However, in the Type IIb gene, whereas the first exon covers only the 5′-UTR, the second exon covers the signal peptide, the complete mature peptide and the 3′-UTR (Figure 2 and Figure S2). All splice sites in both Type II crustin genes followed the canonical GT/AG splicing recognition rule. Interestingly, *L. vannamei* Type IIb genomic organization was quite similar to that found in the crustin*Pm*7, a Type IIb crustin gene from *P. monodon* [18]. By contrast, the *P. monodon* Type IIa crustin*Pm*5 gene [25] showed an exon-intron organization different from that found in the Type IIa gene from *L. vannamei* (Figure 2). Altogether, these results suggest that penaeid Type II crustins are encoded by distinct genomic sequences, and that the molecular diversity found in this crustin sub-group was driven by gene duplication and mutations [26].

### 2.3. Gene Expression Distribution of Type II Crustins in Shrimp Tissues

**Figure 3:**
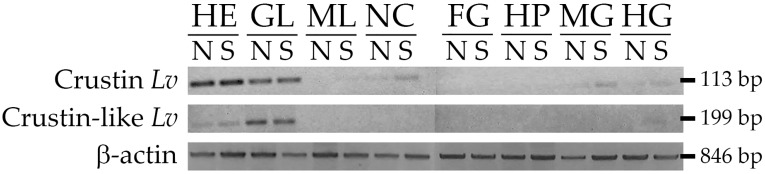
Gene expression distribution of Type II crustins in shrimp tissues. Semiquantitative reverse transcriptase polymerase chain reaction (RT-PCR) analysis of Crustin *Lv* (Type IIa) and Crustin-like *Lv* (Type IIb) transcript levels in different tissues from naïve (N) and *Vibrio*-stimulated (S) shrimp. The expression of the β-actin gene was used as an endogenous control. HE: hemocytes, GL: gills, ML: muscle, NC: nerve cord, FG: foregut, HP: hepatopancreas, MG: midgut, HG: hindgut.

In our transcriptional analyses, we compared the *L. vannamei* Type II crustins in terms of (1) tissue distribution, (2) transcriptional response to two unrelated pathogens, and (3) expression profile during shrimp development. Firstly, the gene expression distribution of Crustin *Lv* (Type IIa) and Crustin-like *Lv* (Type IIb) were evaluated in eight different tissues (hemocytes, gills, muscle, nerve cord, foregut, hepatopancreas, midgut and hindgut) from naïve (unchallenged) animals and shrimp at 48 h post-stimulation with heat-killed bacteria (*V. harveyi*). Interestingly, both *L. vannamei* Type II crustin sub-types showed a similar gene expression distribution in shrimp tissues. Transcripts of Crustin *Lv* and Crustin-like *Lv* were detected in circulating hemocytes and gills of both unchallenged and *Vibrio*-stimulated animals (Figure 3). Remarkably, whereas the highest expression levels of the Crustin *Lv* gene (Type IIa) were found in circulating hemocytes, the Crustin-like *Lv* gene (Type IIb) showed to be predominantly expressed in gills (Figure 3). In *Vibrio*-stimulated animals, the expression of the Crustin *Lv* gene was also detected in the nerve cord and in the two gut portions (midgut and hindgut) (Figure 3). For both Type II crustins, no signals were observed in muscle, foregut, and hepatopancreas (Figure 3). Comparatively, Type II crustins from *P. monodon* were heterogeneously distributed among shrimp tissues. For instance, while crustin*Pm*5 appeared to be constitutively transcribed in the epipodite and eyestalks [25], the other *P. monodon* Type II crustins (crustin*Pm*1, crustin*Pm*4 and crustin*Pm*7) were mainly expressed in hemocytes [10,18,27].

In *L. vannamei*, the expression of Type IIa crustins was restricted to both circulating and tissue-infiltrating hemocytes [28]. More specifically, the expression of this gene was apparently higher in semi-granular hemocytes than in the granular cells [29]. Thus, the detection of Crustin *Lv* transcripts in shrimp tissues was undoubtedly the result of infiltrating hemocytes. By contrast, in this study, Crustin-like *Lv* transcripts were mainly detected in gills. This could be the result of a distinct migratory behavior displayed by circulating hemocytes in shrimp tissues, or the presence of specific tissue-resident hemocyte populations. We cannot rule out the hypothesis of the existence of crustin-expressing hemocytes in shrimp gills distinct from those found circulating in hemolymph. Actually, the presence of tissue-specific subsets of a particular immune cell type has been reported in mammals [30]. Thus, the next import steps will be (1) to colocalize Type II crustins in shrimp hemocytes to verify whether they are expressed in the same hemocyte populations, and (2) to define the precise site of expression of the Crustin-like *Lv* gene by using physical mapping techniques (in situ hybridization and immunohistochemistry) as previously performed for other crustin genes [23,27,28].

### 2.4. Expression Profile of Type II Crustins in Response to Bacterial and Viral Infections

**Figure 4:**
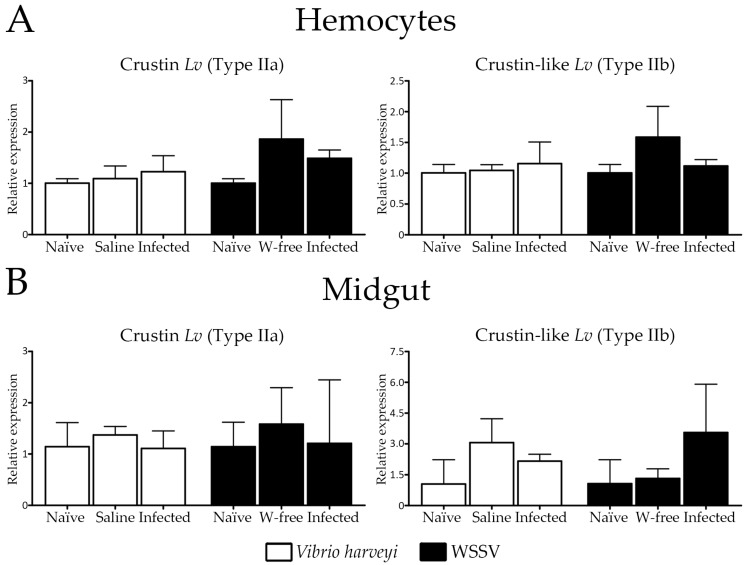
Relative expression profile of Crustin *Lv* (Type IIa) and Crustin-like *Lv* (Type IIb) genes in (**A**) circulating hemocytes and (**B**) midgut of shrimp at 48 h after experimental infections with the Gram-negative *Vibrio harveyi* ATCC 14126 (white bars) or the White spot syndrome virus (black bars). Results are presented as mean ± standard deviation of relative expressions (three biological replicates). WSSV: White spot syndrome virus. W-free: tissue homogenate inoculum prepared from WSSV-free shrimp.

We have further investigated the transcriptional response of Type II crustins in two important immune tissues after experimental infections with the Gram-negative *V. harveyi* and the WSSV. Their expression levels were quantified at 48 h after infections by fluorescence-based reverse transcriptase quantitative PCR (RT-qPCR) in circulating hemocytes and midgut. In a previous study from our group, the expression of gene-encoded AMPs (penaeidins, crustins, anti-lipopolysaccharide factors and stylicins) showed to be modulated in *L. vannamei* shrimp at 48 h post-infections [31]. Importantly, whereas hemocytes are the main site for the expression of AMPs in shrimp [6], the midgut represents an important route of pathogen entry in invertebrates [32]. The transcriptional levels of both Type II crustins were not affected by the bacterial or by the viral infection in circulating hemocytes or in the midgut (Figure 4). Furthermore, no obvious differences in gene expression were observed between unchallenged shrimp and the controls (shrimp injected with SSW or the WSSV-free inoculum) (Figure 4). At this same time point, the expression of the Crustin *Lv* gene (Type IIa) showed to be drastically reduced in circulating hemocytes of shrimp succumbing to a lethal infection by the opportunistic filamentous fungus *Fusarium solani* but not by the WSSV [33]. In our study, the lack of induction of *L. vannamei* Type II crustins could be partly attributed to the time course response of Type II crustins in the analyzed tissues but also to the route of infection. Indeed, we have evaluated the transcriptional response of *L. vannamei* Type II crustins at 48 h post-infections and it is probable that the modulation of these genes has occurred earlier. For instance, in many shrimp species, the expression of crustins showed to be modulated in the first hours post-infections [10,11,12,18]. Moreover, our experimental procedure consisted of injection of the pathogens directly into the shrimp hemocel in order to standardize a same inoculum load per animal [33]. By using a natural route of infection (immersion method), Soonthornchai et al. [34] could observe an increase in crustin expression in the midgut of *P. monodon* shrimp experimentally infected with *V. harveyi*.

Despite the lack of regulation at the transcriptional level, both Type IIa and Type IIb crustins showed to be directly involved in shrimp antimicrobial defense against pathogenic *Vibrio* infections [14,15,16]. Besides, the regulation of these molecules may occur at the post-translational level (maturation and/or trafficking), rather than at the transcriptional level, as observed for penaeidins. Indeed, penaeidins are shrimp gene-encoded AMPs that are constitutively expressed and stored in circulating hemocytes, then released to hemolymph in response to microbial challenges [35]. As with penaeidins, crustins are also produced and stored in hemocytes [23,27], so it is plausible to suppose that they are also released towards the plasma by a regulated secretion pathway triggered by infectious agents.

### 2.5. Expression of Type IIb Crustins during Shrimp Ontogenesis

**Figure 5:**
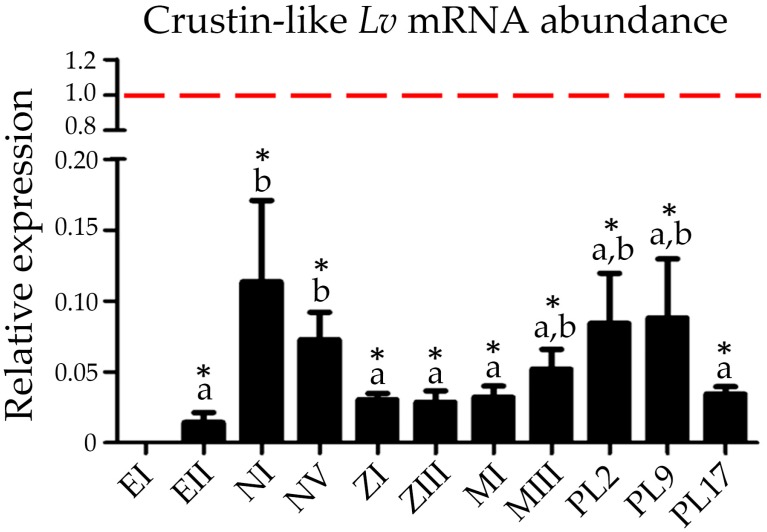
Relative abundance of Crustin-like *Lv* (Type IIb) transcripts during shrimp development. Results are present as mean ± standard deviation (three biological replicates). The red dotted line indicates the basal expression level of the Crustin-like *Lv* gene in hemocytes from juvenile shrimp. EI: fertilized eggs at 0–4 h post-spawning; EII: fertilized eggs at 7–11 h post-spawning; NI: nauplius I; NV: nauplius V; ZI: protozoea I; ZIII: protozoea III; MI: mysis I; MIII: mysis III; PL2: postlarva 2; PL9: postlarva 9; PL17: postlarva 17. Different letters indicate significant differences among the developmental stages from EII to PL17 (one-way ANOVA/Tukey, *p* < 0.05). Asterisks (*) shows significant differences between each developmental stage and hemocytes from juveniles (one-way ANOVA/Tukey, *p* < 0.05).

Another important result from this study was the first characterization of the transcriptional profile of a Type IIb crustin during shrimp development. The transcript abundance of the Crustin-like *Lv* gene (Type IIb) was quantified by RT-qPCR in the 12 developmental stages of *L. vannamei*, from fertilized eggs to larval and postlarval stages, and also in circulating hemocytes from juveniles. Crustin-like *Lv* expression was detected in all shrimp developmental stages, but only quantified from fertilized eggs at 7–11 h post-spawning (Figure 5). Interestingly, Crustin-like *Lv* gene expression profile during shrimp ontogenesis was quite similar to that which was previously observed for the Crustin *Lv* gene [36]. Both Type II crustins were transcribed at variable levels during shrimp development. However, the highest mRNA levels were observed in hemocytes from juveniles (Figure 5), which is in accordance with our results of tissue expression distribution (Figure 3). Moreover, transcripts for both genes were found in fertilized eggs, revealing a maternal contribution of these AMPs to the shrimp offspring [36]. Interestingly, Suleiman et al. [23] detected the presence of the carcinin antimicrobial peptide (Type I crustin) at protein levels in the ovaries and oocytes of the shore crab *C. maenas*. The expression of crustins in early larval stages of *L. vannamei* suggests the participation of gene-encoded AMPs in a critical phase where the immune system has not been fully developed. Therefore, the specific role of Type II crustins during shrimp development claims for further investigations.

### 2.6. Molecular Signatures for Type II Crustins in Penaeid Shrimp

**Figure 6:**
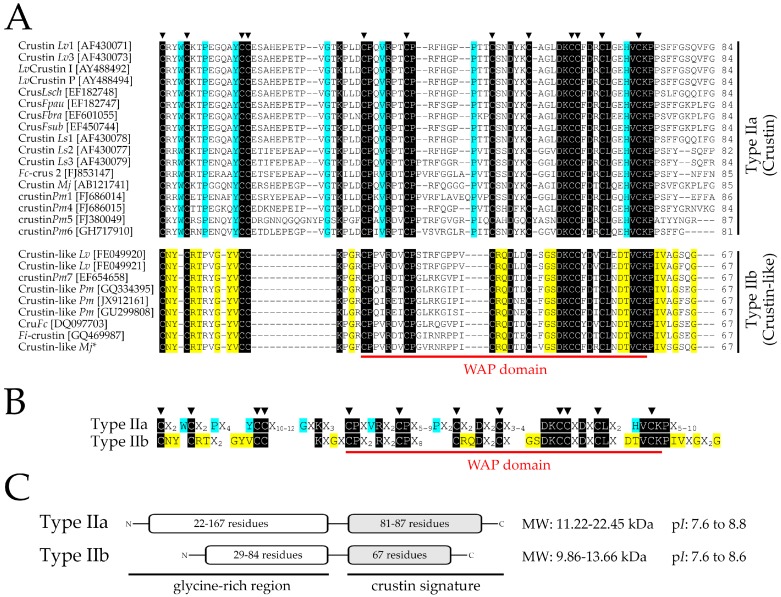
The molecular signature of shrimp Type II crustins. (**A**) Amino acid sequence alignments of the C-terminal region holding the 12 conserved cysteine residues (the crustin signature [39]) of Type IIa and Type IIb crustins from penaeid shrimp: *Litopenaeus vannamei* (Crustin *Lv*1, Crustin *Lv*3, *Lv*Crustin I, *Lv*Crustin P, Crustin-like *Lv*), *Litopenaeus setiferus* (Crustin *Ls*1, Crustin *Ls*2, Crustin *Ls*3), *Litopenaeus schmitti* (Crus*Lsch*), *Farfantepenaeus paulensis* (Crus*Fpau*), *Farfantepenaeus brasiliensis* (Crus*Fbra*), *Farfantepenaeus subtilis* (Crus*Fsub*), *Fenneropenaeus chinensis* (*Fc*-crus 2, Cru*Fc*), *Fenneropenaeus indicus* (*Fi*-crustin), *Penaeus monodon* (crustin*Pm*1, crustin*Pm*4, crustin*Pm*5, crustin*Pm*6, crustin*Pm*7, Crustin-like *Pm*), and *Marsupenaeus japonicus* (Crustin *Mj*, Crustin-like *Mj*). (**B**) Consensus amino acid sequence of shrimp Type IIa and Type IIb crustins. X indicates any amino acid. Identical residues are highlighted in black. Specific amino acid residues conserved in shrimp Type IIa and Type IIb crustins are highlighted in blue and yellow, respectively. Triangles (▼) indicate the 12 conserved cysteine residues found in crustins. The whey acidic protein (WAP) domain is underlined by a solid red line. (**C**) A not-to-scale representation of shrimp Type IIa and Type IIb crustins indicating the N-terminal glycine-rich region and the C-terminal crustin signature (cysteine-rich region + WAP domain). MW: molecular weight. p*I*: theoretical isoelectric point. * Sequence obtained from [16] (not deposited in any database).

**Figure 7:**
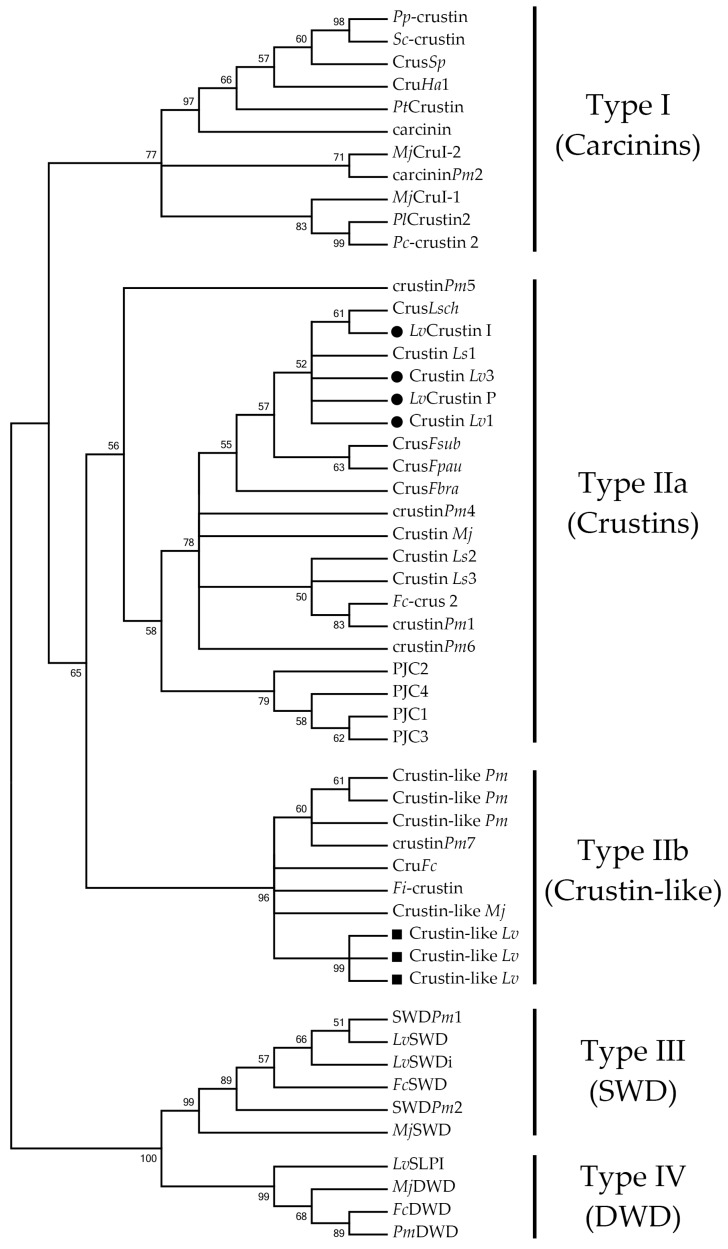
Penaeid shrimp Type II crustins form two distinct phylogenetic clades. The phylogenetic tree was constructed using the Neighbor-Joining method in MEGA 6. Bootstrap sampling was reiterated 1000 times. Sequences included in analyses were the following: (i) Type I crustins (“Carcinins”): the *Pp*-crustin from *Portunus pelagicus* (GenBank: JQ965930), the *Sc*-crustin from *Scylla serrata* (GenBank: HQ638025), the Crus*Sp* from *Scylla paramamosain* (GenBank: EU161287), the Cru*Ha*1 from *Hyas araneus* (GenBank: EU921641), *Pt*Crustin from *Portunus trituberculatus* (GenBank: FJ612106), the carcinin (11.5-kDa peptide) from *Carcinus maenas* (GenBank: AJ427538), the *Pl*Crustin2 from *Pacifastacus leniusculus* (GenBank: EF523613), the *Pc*-crustin 2 from *Procambarus clarkii* (GenBank: GQ301202), the *Mj*CruI-1 and *Mj*CruI-2 from *Marsupenaeus japonicus* [11], and the carcinin*Pm*2 from *Penaeus monodon* [10]; (ii) Type IIa crustins (“Crustins”): the *Lv*Crustin I (GenBank: AY488492), *Lv*Crustin P (GenBank: AY488494), Crustin *Lv*1 (GenBank: AF430071) and Crustin *Lv*3 (GenBank: AF430073) from *Litopenaeus vannamei* (indicated by black circles, ●), the Crustin *Ls*1 (GenBank: AF430078), Crustin *Ls*2 (GenBank: AF430077) and Crustin *Ls*3 (GenBank: AF430079) from *Litopenaeus setiferus*, the Crus*Lsch* (GenBank: EF182748) from *Litopenaeus schmitti*, the Crus*Fpau* (GenBank: EF182747) from *Farfantepenaeus paulensis*, the Crus*Fbra* (GenBank: EF601055) from *Farfantepenaeus brasiliensis*, the Crus*Fsub* (GenBank: EF450744) from *Farfantepenaeus subtilis*, the Crustin *Mj* (GenBank: AB121741) from *M. japonicus*, the crustin*Pm*1 (GenBank: FJ686014), crustin*Pm*4 (GenBank: FJ686015), crustin*Pm*5 (GenBank: FJ380049) and crustin*Pm*6 (GenBank: GH717910) from *P. monodon*, the *Fc*-crus 2 (GenBank: FJ853147) from *Fenneropenaeus chinensis* and the PJC1 (GenBank: FJ797417), PJC2 (GenBank: FJ797418), PJC3 (GenBank: FJ797419), and PJC4 (GenBank: FJ797420) from *Panulirus japonicus*; (iii) Type IIb crustins (“Crustin-like”): the Crustin-like *Lv* (GenBank: JQ824114, FE049920 and FE049921) from *L. vannamei* (indicated by black squares, ■), the Crustin-like *Mj* from *M. japonicus* [16], the Cru*Fc* (GenBank: DQ097703) from *F. chinensis*, the *Fi*-crustin (GenBank: GQ469987) from *Fenneropenaeus indicus* and the Crustin-like *Pm* (GenBank: GQ334395, JX912161 and GU299808) and crustin*Pm*7 (GenBank: EF654658) from *P. monodon*; (iv) Type III crustins (“Single WAP domain-containing proteins or SWD”): the *Lv*SWD (GenBank: AY464465) and *Lv*SWDi [40] from *L. vannamei*, the SWD*Pm*1 (GenBank: EU623979) and SWDPm2 (GenBank: EU623980) from *P. monodon*, the *Fc*SWD (GenBank: EF216349) from *F. chinensis* and the *Mj*SWD (GenBank: AU176270) from *M. japonicus*; (v) Type IV crustins (“Double WAP domain-containing proteins or DWD”): the *Lv*SLPI (GenBank: EF467169) from *L. vannamei*, the *Pm*DWD (GenBank: BI784457) from *P. monodon*, the *Fc*DWD (GenBank: GQ303571) from *F. chinensis*, and the *Mj*DWD (GenBank: EU095018) from *M. japonicus*.

With the comparative transcriptional profiling of Crustin *Lv* and Crustin-like *Lv* genes in hand, we focused our attention on the molecular diversity of Type II crustins in penaeid shrimp (Decapoda: Penaeidae). Full coding sequences of Type II crustins were systematically collected from both annotated (GenBank Nucleotide database) and non-annotated (EST and TSA libraries) nucleotide databases and used for multiple-sequence alignments (Figure 6 and Figure S1) and phylogenetic reconstructions (Figure 7). An important contribution to this analysis was undoubtedly the identification of Crustin-like sequences in an Occidental penaeid species, *L. vannamei*, since Type IIb crustins have been only reported in the Asian shrimps *P. monodon*, *F. chinensis*, *F. indicus* and *M. japonicus* [16,17,18,22]. However, our in silico mapping method failed to recover Type IIb crustin sequences in other penaeid shrimp from the Western Hemisphere (as well as shrimp from other genera), due to the lack of genomic and transcriptomic data available for those species. Moreover, it is important to point out the confused nomenclature adopted in the literature concerning the use of the terms “crustin” and “crustin-like peptides” [15,37,38], evidencing that the terminology of this AMP family need to be fully revised.

The striking information given by the sequence analysis was the identification of amino acid signatures specific to Type IIa and Type IIb crustins in penaeid shrimp. We found a molecular pattern based on conserved amino acid residues that can successfully discriminate Type IIa and Type IIb crustins at the primary structure level (Figure 6). The classification of Type II crustins into two sub-groups (Type IIa and IIb) was initially proposed by Tassanakajon et al. [13], that is essentially based on differences in the amino acid length of the glycine-rich region and on the distance between the cysteine-rich region and the WAP domain. By contrast, multiple alignments of the amino acid sequences of all available shrimp Type II crustins revealed that the differences between Type IIa and Type IIb crustins lie in (1) the presence of specific amino acid residues positioned at precise locations of the C-terminal region holding the cysteine-rich region and the WAP domain, and (2) the arrangement (amino acid intervals) of the 12 conserved cysteine residues (Figure 6A,B). These 12 conserved cysteine residues (four from the cysteine-rich region and eight from the WAP domain) comprise a region previously termed as “crustin domain” (the crustin signature) [39] that is exclusively found in Type I (carcinins) and Type II (crustins and crustin-like peptides) crustins. On the other hand, Type III and Type IV crustins possess one and two WAP domains, respectively, but not the cysteine-rich region found in Type I and Type II crustins [5]. Thus, both Type III (SWD) and Type IV (DWD) crustins lack the crustin signature proposed by Zhao and Wang [39].

Type IIa crustins from penaeid shrimp contain three amino acid residues between the first two cysteine residues of the crustin signature (Cys_1_-Cys_2_) within a conserved tryptophan (Trp or W) preceding Cys_2_. Additionally, a sequence of 16–18 amino acids in length is found between Cys_4_-Cys_5_ and a sequence of 8–12 residues between Cys_6_-Cys_7_ (Figure 6A). Comparatively, Type IIb crustins contain an asparagine (Asn or N) and a tyrosin (Tyr or Y) between Cys_1_-Cys_2_ and only four residues between Cys_4_-Cys_5_ (Lys-Pro/Leu-Gly-Arg/Phe) (Figure 6A). Moreover, all Type IIb crustins contain a sequence of eight residues between Cys_6_-Cys_7_ and characteristic amino acid residues in the WAP domain that are not found in Type IIa crustins (Figure 6A). The specific amino acid signature of each sub-group of shrimp Type II crustins is presented in Figure 6B. Finally, whereas the N-terminal glycine-rich region of Type IIb crustins are composed of 29–84 amino acid residues, the glycine-rich region of Type IIa crustins can reach up to 167 residues in length (e.g., crustin*Pm*4 from *P. monodon* [10]). On the other hand, whereas the amino acid length of the C-terminal region of Type IIa crustins showed to be variable (81–87 residues), the C-terminal region (holding the crustin signature) of all Type IIb crustins is composed of 67 amino acid residues (Figure 6C). Despite their differences in terms of size and molecular weight, shrimp Type II crustins are cationic peptides/polypeptides with theoretical isoelectric point (p*I*) ranging from 7.6 to 8.8.

Phylogenetic reconstructions clearly supported the distinction of shrimp Type II crustins into two separate groups. The obtained phylogenetic trees first showed that the four crustin Types found in crustaceans (Types I to IV) clustered into two distinct main clades: a first clade covering both Type I and Type II crustins and a second clade containing the sequences that lack the crustin signature (Type III and Type IV crustins) (Figure 7). Regarding the first clade, Type I and Type II crustins were split into distinct groups. Within the Type II group, shrimp Type IIa crustins (Dendrobranchiata) and crustins from the spiny lobster *P. japonicus* (Pleocyemata) formed a separate clade from Type IIb crustins (Figure 7). Indeed, as shrimp Type II crustins, *P. japonicus* crustins (PJC1 to PJC4) also harbor a portion rich in glycine residues at the N-terminal region [24]. From our analysis, *P. japonicus* crustins correspond to authentic Type II crustins since they hold the conserved amino acid residues found in the Sub-type IIa (Figure 6B). Moreover, while Type IIa crustins showed to be present in different decapod groups (Pleocyemata and Dendrobranchiata), Type IIb crustins appear to be exclusive of penaeid shrimp (Dendrobranchiata). Interestingly, the Type IIa crustin*Pm*5 from *P. monodon* formed a distinct clade from the other Type IIa crustins from penaeid shrimp (Dendrobranchiata) and *P. japonicus* (Pleocyemata). Indeed, crustin*Pm*5 is a unique crustin member within the Type IIa clade, apparently exclusive of the black tiger shrimp *P. monodon*, that is diverse not only in terms of sequence, but also in terms of genomic organization and tissue expression distribution [25]. Both sequence and phylogenetic data showed that Type IIa crustins display a high degree of diversity at both inter- and intraspecific levels when compared to Type IIb crustins. For instance, Type IIb crustins from distant shrimp species have been shown to be more similar to each other than Type IIa crustins from the same shrimp. This is the case of *P. monodon* Type IIa crustins that are diverse not only in terms of sequence, but also in genomic organization and gene expression regulation [10,27].

Why Type IIb crustins are quite similar among distant shrimp species is still an open question. On one hand, Type IIb crustins could be recently evolved from an ancestor shrimp Type II gene and, on the other hand, the interspecific conservation of Type IIb crustin sequences could be the result of selective pressures (environmental stressors, pathogens, etc.). In any cases, phylogenetic and maximum likelihood-based codon substitution analyses have shown that diversity in crustin family has been driven by successive gene duplications and positive Darwinian selection [26]. Our transcriptional data revealed that *L. vannamei* Type II crustins display a very similar pattern of gene expression in terms of tissue distribution, transcriptional response to pathogens, and mRNA abundance during shrimp development. Taken together, it is plausive to hypothesize that Type IIa and Type IIb crustins evolved by gene duplication from a common ancestral gene that conserved a similar pattern of transcriptional regulation. The apparent lack of Type IIb crustin sequences in available databases of other crustacean groups suggests that the gene duplication event relative to Type IIa–Type IIb divergence occurred exclusively in the Dendrobranchiata lineage.

## 3. Materials and Methods

### 3.1. Sequence Data Analysis and Phylogeny

Annotated Type IIb sequences (Crustin-like peptides) were systematically collected from publicly accessible databases and used for the search of homologous sequences in *L. vannamei* annotated (GenBank Nucleotide) and non-annotated EST (Expressed Sequence Tags) and TSA (Transcriptome Shotgun Assembly) databases. The complete nucleotide sequence of the Type II crustin genes was obtained by mining the genome of *L. vannamei* [41]. Homology searches were performed using BLAST at NCBI. All nucleotide sequences were manually inspected and translated using the ExPASy Translate Tool (http://web.expasy.org/translate/).

Prediction of signal peptide was performed with the SignalP 4.1 program (http://www.cbs.dtu.dk/services/SignalP/) and the theoretical isoelectric point (p*I*) and molecular weight (MW) of the mature peptides were predicted using the ExPASy ProtParam Tool (http://web.expasy.org/protparam/). Multiple alignments of the deduced amino acid sequences were generated using the BioEdit version 7.0 sequence alignment editor (http://www.mbio.ncsu.edu/BioEdit/bioedit.html). Phylogenetic analysis based on the amino acid sequences of crustins from different crustaceans were conducted in MEGA version 6.0 [41] using the Neighbor-Joining method (complete deletion option). Trees were resampled 1000 times.

### 3.2. Animals, Microbial Stimulation and Tissue Collection

*Litopenaeus vannamei* juveniles (10 ± 2 g) were obtained from the Laboratory of Marine Shrimps (Federal University of Santa Catarina, Brazil). Following acclimation (one week), five animals were injected with 5 × 10^7^ colony-forming units (CFU)/animal of heat-killed (70 °C for 20 min) *Vibrio harveyi* ATCC 14126 under 100 µL sterile seawater (SSW). Unchallenged shrimp (*n* = 5) were used as control. At 48 h post-stimulation, circulating hemocytes were obtained as previously described [33] and shrimp organs (gills, muscle, nerve cord, foregut, hepatopancreas, midgut and hindgut) were harvested by dissection. Tissue samples were washed in a Tris-saline solution (10 mM Tris, 330 mM NaCl, pH 7.4), homogenized in TRIzol reagent (Thermo Fisher Scientific, Waltham, MA, USA) and immediately processed for RNA isolation and tissue distribution analysis.

### 3.3. Experimental Infections

The experimental infections were performed with two shrimp pathogens, the Gram-negative *Vibrio harveyi* ATCC 14126 and the White spot syndrome virus (WSSV). For the bacterial infection, shrimp were injected with 6 × 10^7^ CFU/animal of live *V. harveyi* ATCC 14126 (under 100 µL SSW) or with 100 µL SSW (aseptic injury control). For the viral infection, shrimp were injected with 100 µL of a WSSV inoculum containing 3 × 10^2^ viral particles. The WSSV inoculum was prepared as previously described [33]. Animals injected with 100 µL of a tissue homogenate prepared from WSSV-free shrimp were used as injury control for the viral infection. At 48 h post-infections, circulating hemocytes and midguts were collected and separately pooled (three pools of five animals per condition) for total RNA isolation and fluorescence-based RT-qPCR analysis. Unchallenged animals (naïve shrimp at time 0 h) were used as control for all experimental conditions.

### 3.4. Reverse Transcription-Polymerase Chain Reaction (RT-PCR) Analysis for Tissue Distribution of Gene Expression

Total RNA was purified using TRIzol reagent (Thermo Fisher Scientific) according to manufacturer’s specifications, treated with DNaseI (Thermo Fisher Scientific) for 15 min at 37 °C to remove remaining genomic DNA and precipitated with 0.3 M sodium acetate (pH 5.2) and isopropanol (1:1; v:v). First strand cDNA was synthesized from 1 µg of total RNA using RevertAid Reverse Transcriptase (Thermo Fisher Scientific) and oligo(dT)_12–18_ primers. PCR reactions were conducted in a 15-µL reaction volume using 1 µL of synthesized cDNA (diluted 1:10) as template. The primer sequences are listed in Table 1. The PCR conditions were as follows: 1 cycle of denaturation at 95 °C for 10 min followed by 40 cycles of 95 °C for 45 s, 60 °C for 45 s and 72 °C for 45 s, and a final extension step of 72 °C for 7 min. The PCR products were analyzed by electrophoresis in a 1.5% agarose and stained by ethidium bromide. The expression of the β-actin gene was used to normalize the RT-PCR data for comparison.

### 3.5. Fluorescence-Based Reverse Transcription Real-Time Quantitative PCR (RT-qPCR)

RT-qPCR amplifications were performed using the StepOne Plus Real-time PCR System (Thermo Fisher Scientific) in a final volume of 15 µL containing 0.3 µM of each primer, 7.5 µL of reaction mix (Maxima SYBR Green/ROX qPCR Master Mix 2×; Thermo Fisher Scientific) and 1 µL of diluted cDNA (1:20). Primer sequences are listed in Table 1. The PCR conditions were as follow: 95 °C for 10 min, followed by 40 cycles of 95 °C for 15 s and 60 °C for 1 min. Melt curve analysis (60–95 °C at a temperature transition rate of 0.05 °C/s) for each primer pair was performed to ensure primer specificity with continuous fluorescence acquisition The efficiency of amplification was determined with a standard curve using a five-point dilution curve of a pool of cDNA samples. The eukaryotic translation elongation factor 1-alpha (*Lv*EF1α) and the ribosomal proteins *Lv*RpS6, *Lv*L40 and *Lv*RpS3A were used as reference genes for RT-qPCR data normalization using the 2^−ΔΔCq^ method [42]. Statistical significance was considered at *p* < 0.05 by one-way ANOVA followed by Tukey’s multiple comparison test.

### 3.6. Quantitative Gene Expression Analysis during Shrimp Development

Three biological replicates of 12 development stages of *L. vannamei* were collected: fertilized eggs at 0–4 h (EI) and at 7–11 h post-spawning (EII), nauplius I and V (NI and NV), protozoea I and III (ZI and ZIII), mysis I and III (MI and MIII) and postlarvae aged of 2, 9 and 17 days (PL2, PL9 and PL17). Crustin-like *Lv* transcript levels during shrimp development were quantified by RT-qPCR and normalized with the gene expression of *Lv*RpS6 and β-actin, as previously described [36]. Hemocyte samples from juvenile shrimp (three pools of five animals) were used as control for calibrating gene expression data. Statistical significance was considered at *p* < 0.05 by one-way ANOVA followed by Tukey’s multiple comparison test.

## 4. Conclusions

In conclusion, we have identified for the first time a Type IIb crustin (Crustin-like *Lv*) in an Occidental (non-Asian) penaeid, the Pacific white shrimp *L. vannamei*. The molecular characterization and comparison of Crustin-like *Lv* with other crustin sequences allowed to the identification of molecular amino acid signatures specific to Type II crustins in penaeid shrimp. Despite their similar patterns of gene expression, Type IIa and Type IIb form two distinct groups of crustins in penaeid shrimp with potential specific biological activities. Results from the in silico and phylogenetic analyses combined with our gene expression data suggested that shrimp Type II crustins evolved from a common ancestral gene that conserved a similar pattern of transcriptional regulation. The functional implication of the molecular diversity of Type II crustins in the shrimp immune response is an important task to be assessed by reverse genetic approaches. These bioactive molecules from marine invertebrates could find applications not only in aquaculture and shrimp farming, but also in both human and veterinary medicine.

## Figures and Tables

**Table 1:** Nucleotide sequences of primers used in this study.

Gene	Forward Primer (5′-3′)	Reverse Primer (5′-3′)	Amplicon
*Primers for Tissue Distribution Analysis (RT-PCR)*			
β-actin	TAATCCACATCTGCTGGAAGGTGG	TCACCAACTGGGATGACATGG	846 bp
Crustin *Lv*	CGAACCAGAGACACCTGTTG	CAGCACACTTGTAGTCGTTG	113 bp
Crustin-like *Lv*	GCAGGATAAAGACAAGGC	GTAATTGCAGTTGAATCCGCC	199 bp
*Primers for Quantitative Analysis of Gene Expression (RT-qPCR)*			
*Lv*EF1α	TGGCTGTGAACAAGATGGACA	TTGTAGCCCACCTTCTTGACG	103 bp
*Lv*RpS6	AGCAGATACCCTTGGTGAAG	GATGCAACCACGGACTGAC	193 bp
*Lv*L40	GAGAATGTGAAGGCCAAGATC	TCAGAGAGAGTGCGACCATC	104 bp
*Lv*RpS3A	GGCTTGCTATGGTGTGCTCC	TCATGCTCTTGGCTCGCTG	101 bp
β-actin	CCACGAGACCACCTACAAC	AGCGAGGGCAGTGATTTC	142 bp
Crustin *Lv*	CGAACCAGAGACACCTGTTG	CAGCACACTTGTAGTCGTTG	113 bp
Crustin-like *Lv*	GCAGGATAAAGACAAGGC	GTAATTGCAGTTGAATCCGCC	199 bp

## Supplementary Materials

The following are available online at www.mdpi.com/1660-3397/16/1/31/s1, Figure S1: Amino acid sequence alignments of shrimp Type II crustins: *Litopenaeus vannamei* (*Lv*Crustin P, *Lv*Crustin I, Crustin *Lv*1, Crustin *Lv*3, Crustin-like *Lv*), *Litopenaeus setiferus* (Crustin *Ls*1, Crustin *Ls*2, Crustin *Ls*3), *Farfantepenaeus paulensis* (Crus*Fpau*), *Farfantepenaeus brasiliensis* (Crus*Fbra*), *Farfantepenaeus subtilis* (Crus*Fsub*), *Litopenaeus schmitti* (Crus*Lsch*), *Marsupenaeus japonicus* (Crustin *Mj*, Crustin-like *Mj*), *Fenneropenaeus chinensis* (*Fc*-crus 2, Cru*Fc*), *Penaeus monodon* (crustin*Pm*1, crustin*Pm*4, crustin*Pm*5, crustin*Pm*6, crustin*Pm*7, Crustin-like *Pm*) and *Fenneropenaeus indicus* (*Fi*-crustin). The predicted signal peptides are in bold and underlined. Identical amino acid residues are shadowed with black backgrounds. Triangles (▼) indicate the 12 conserved cysteine residues found in crustins. * Sequence obtained from [16] (not deposited in any database). Figure S2: Genomic nucleotide and deduced amino acid sequences of Type IIa Crustin *Lv* (**a**) and Type IIb Crustin-like *Lv* (**b**). The predicted signal peptides are in bold and underlined. The exon and intron sequences are shown in black and grey, respectively. A dash (-) marks the stop codon.
