# Supplementary material for: Specific Molecular Signatures for Type II Crustins in Penaeid Shrimp Uncovered by the Identification of Crustin-Like Antimicrobial Peptides in Litopenaeus vannamei
Source: Mar Drugs. 2018 Jan 16;16(1):31. doi: 10.3390/md16010031 (PMC5793079; doi:10.3390/md16010031)
Supplement: Supplementary file 1 [file marinedrugs-16-00031-s001.pdf]

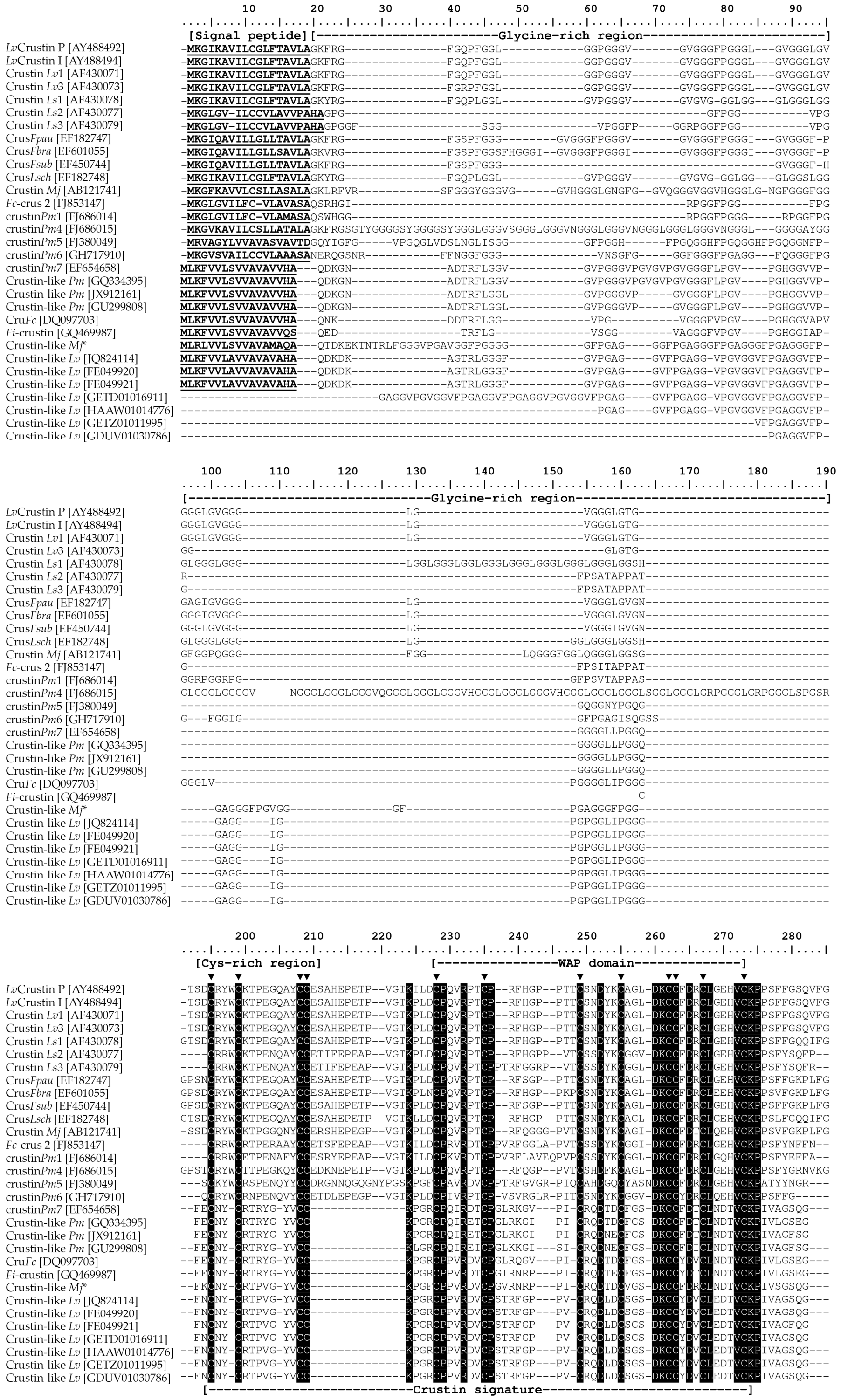

*Sun et al. *Fish Shellfish Immunol*. 2017 Jul;66:487-496.

Figure S1. Amino acid sequence alignments of shrimp Type II crustins: *Litopenaeus vannamei* (*Lv*Crustin P, *Lv*Crustin I, Crustin *Lv*1, Crustin *Lv*3, Crustin-like *Lv*), *Litopenaeus setiferus* (Crustin *Ls*1, Crustin *Ls*2, Crustin *Ls*3), *Farfantepenaeus paulensis* (Crus*Fpau*), *Farfantepenaeus brasiliensis* (Crus*Fbra*), *Farfantepenaeus subtilis* (Crus*Fsub*), *Litopenaeus schmitti* (Crus*Lsch*), *Marsupenaeus japonicus* (Crustin *Mj*, Crustin-like *Mj*), *Fenneropenaeus chinensis* (*Fc*-crus 2, Cru*Fc*), *Penaeus monodon* (crustin*Pm*1, crustin*Pm*4, crustin*Pm*5, crustin*Pm*6, crustin*Pm*7, Crustin-like *Pm*) and *Fenneropenaeus indicus* (*Fi*-crustin). The predicted signal peptides are in bold and underlined. Identical amino acid residues are shadowed with black backgrounds. Triangles (▼) indicate the 12 conserved cysteine residues found in crustins. * Sequence obtained from [16] (not deposited in any database).

A

Crustin *Lv* gene (Type IIa)

```
GCACACCACTAGCTTGTACTGGAGGCAACCATGAAGGTATGAGCTTGTGTTTGTTTTCGT   60
                              M  K
TGACTTCCGGTCTTTTTTTTTTTTTTTTTTCTCTCTTTTTTTTTAAGTGAGAGAGTTTAA  120
TGTAAGGAGTTTTTTTTAATGTTTTTTTCTTTGTGTACACCGTTTAAATATCATGTTTTG  180
AGTCATGATATATACAAATGCGAGATAGTTATTGTTGTGAATGTACTTCCCCTTTCGCGG  240
GATATTTATAGATTTGACTATCAAATGTGCATTTCGGATTAAAAAAGATTTTTAGTCCAA  300
AATGCAAACTTCGGTGTTAAATCTATAAATAGTATTATTGCGTGATGGTAACGTAAATAC  360
ACTAAAGATTAAAGCTTATAATCATTCAATTTTGCTATGTCTTCGCCACGAAAATTTGCA  420
ACGAAAATGTCATATACAAACATTTTAGGCTATGCACTGATGTTAGGCATACATATTATT  480
GTGCATAGCCAGGTTGGATAAAAACGTTTACAAGATGAAAGGCTTAATCTAGATCCAAGC  540
TATTACGGTACAATATATTCCGCATTTCGAAAGGTGATTAAAAAGAGATTTCATTATTGA  600
ATGCAAAAGACCATCACACAGGTATTATGGCTTTCTCTCTGTATACAGCATTTTTTTCCC  660
TAAAGCTTCCCTAAAATACCGACGTTACGGTAACAGTAACGTTAAGCAATATAGATTGAT  720
ATAGAACAAATCGAAATGACGGAAGTTGATATATAACAATAACACGATAAAGATTGTTTG  780
TCTTAAGAATAATCTTTCTCCCGTTTGTCCCACTGCTCAGACTTATAAAAAAGCCATTGG  840
ACTTTCTCAACAATACAGCAGGGACTCCTTTAACACTGACAACGTTTCCGAACAATGCAA  900
TGACTTTAATTTCTATTTTACACCAGAGTAATTTCTATTTTTAATGCTATTTTTCAATCG  960
TTCTCTTTCAGGGCATCAAGGCGGTGATTCTGTGCGGCCTCTTTACGGCGGTTTTGGCTG 1020
            G  I  K  A  V  I  L  C  G  L  F  T  A  V  L  A
GCAAGTTTCGCGGCTTCGGACAGCCATTTGGAGGTCTGGGTGGTCCAGGAGGCAGTGTAG 1080
G  K  F  R  G  F  G  Q  P  F  G  G  L  G  G  P  G  G  S  V
GTGTTGGTGGTGGTTTCCCCGGAGGCGGTTTAGGTGTAGGTGGTGGTCTTGGTGTAGGTG 1140
G  V  G  G  G  F  P  G  G  G  L  G  V  G  G  G  L  G  V  G
GCGGTCTTGGTGTAGGTGGCGGTCTTGGTGTAGGTGGCGGTCTTGGAACTGGCACAAGCG 1200
G  G  L  G  V  G  G  G  L  G  V  G  G  G  L  G  T  G  T  S
ACTGCAGGTATTGGTGCAAGACTCCGGAGGGTCAAGCCTACTGCTGCGAGTCGGCCCACG 1260
D  C  R  Y  W  C  K  T  P  E  G  Q  A  Y  C  C  E  S  A  H
AACCAGAGACACCTGTTGGCACCAAGCCACTCGACTGCCCACAAGTCCGTCCCACATGCC 1320
E  P  E  T  P  V  G  T  K  P  L  D  C  P  Q  V  R  P  T  C
CACGTTTCCATGGGCCCCCCACAACCTGTTCCAACGACTACAAGTGTGCTGGCCTCGATA 1380
P  R  F  H  G  P  P  T  T  C  S  N  D  Y  K  C  A  G  L  D
AGTGTTGCTTCGACAGGTGTTTGGGAGAACACGTGTGCAAGCCTCCCTCATTCTTCGGAT 1440
K  C  C  F  D  R  C  L  G  E  H  V  C  K  P  P  S  F  F  G
CGCAGGTTTTCGGATGAAGAATAAGCACGAAAGAATTTGAAAGGATGAAGAGAAAGAAGA 1500
S  Q  V  F  G  -
AAAGACCATCTGAAGAACGACCGATGTTTTGGAATTTGACTGAAAAAAGAAAGAAAAACA 1560
GGGAATTCTTTCTTTCTGTAGGATTTATCTGATTAACATGATTTTTGTTATATGTGAATT 1620
AGACTATTCTTCTGTCAAAAGAAACTTATAG                              1651
```

B

Crustin-like *Lv* gene (Type IIb)

```
CAGTCCGTTCGTCGCAGAGCAAGGAGAAGTATTACAAAGGTGAGGAGGAGAAGCCAACAT  60
TACGAGGCTATGCTTTGAGTTACCAGCTTTGGTTGTCCAACTGTTTCTTTAAAGTCATAG 120
AAACTCTTACCAATGATCGTGTCAATCACTAGGAGACAAAATGCTGTCCTGTGACAATTC 180
AAAGCTCAAATGTCACTCCATGATCTTAATTCCGCGTTCGTTTCTTCCTTGTAGATGCTG 240
                                                       M  L
AAGTTTGTAGTATTAGCCGTTGTCGCCGTGGCCGTGGCGCACGCGCAGGATAAAGACAAG 300
 K  F  V  V  L  A  V  V  A  V  A  V  A  H  A  Q  D  K  D  K
GCCGGCACTCGCTTAGGAGGAGGATTCGGGGTTCCTGGAGCCGGTGGCGTCTTCCCAGGA 360
 A  G  T  R  L  G  G  G  F  G  V  P  G  A  G  G  V  F  P  G
GCCGGTGGCGTCCCTGGAGTAGGTGGCGTCTTTCCTGGAGCCGGTGGCGTCTTCCCTGGA 420
 A  G  G  V  P  G  V  G  G  V  F  P  G  A  G  G  V  F  P  G
GCCGGTGGTATCGGTCCTGGACCCGGCGGCCTCATCCCCGGAGGCGGATTCAACTGCAAT 480
 A  G  G  I  G  P  G  P  G  G  L  I  P  G  G  G  F  N  C  N
TACTGCAGGACGCCCGTCGGGTACGTCTGCTGCAAGCCCGGTAGGTGCCCTCCGGTTCGA 540
 Y  C  R  T  P  V  G  Y  V  C  C  K  P  G  R  C  P  P  V  R
GACGTCTGCCCGTCGACCCGCTTCGGACCCCCGGTCTGCCGCCAGGACCTGGACTGCTCC 600
 D  V  C  P  S  T  R  F  G  P  P  V  C  R  Q  D  L  D  C  S
GGCTCCGACAAGTGCTGCTATGACGTCTGCCTGGAAGACACAGTCTGCAAACCCATCGTG 660
 G  S  D  K  C  C  Y  D  V  C  L  E  D  T  V  C  K  P  I  V
GCAGGTTCTCAGGGATAAGCCTGCATGTGAAACTTATCAAGCCTTCGTTATCAAATAAAT 720
 A  G  S  Q  G  -
GCTATAACTGTTAATTGTAAAT                                       742
```

Figure S2. Genomic nucleotide and deduced amino acid sequences of Type IIa Crustin *Lv* (**A**) and Type IIb Crustin-like *Lv* (**B**). The predicted signal peptides are in bold and underlined. The exon and intron sequences are shown in black and grey, respectively. A dash (-) marks the stop codon.
